# An evolutionary perspective on the relationship between kinetochore size and CENP-E dependence for chromosome alignment

**DOI:** 10.1242/jcs.263466

**Published:** 2024-12-19

**Authors:** Ana C. Almeida, Helder Rocha, Maximilian W. D. Raas, Hanh Witte, Ralf J. Sommer, Berend Snel, Geert J. P. L. Kops, Reto Gassmann, Helder Maiato

**Affiliations:** ^1^ i3S - Instituto de Investigação e Inovação em Saúde, Universidade do Porto, Rua Alfredo Allen 208, 400-135 Porto, Portugal; ^2^ Instituto de Biologia Molecular e Celular, Universidade do Porto, Rua Alfredo Allen 208, 400-135 Porto, Portugal; ^3^ Escola Superior de Saúde, Instituto Politécnico do Porto, Rua Dr. António Bernardino de Almeida, 420-075 Porto, Portugal; ^4^ Oncode Institute, Hubrecht Institute – KNAW, and University Medical Center Utrecht, 3584 CT, Utrecht, Netherlands; ^5^ Theoretical Biology and Bioinformatics, Department of Biology, Faculty of Science, Utrecht University, 384 CH Utrecht, the Netherlands; ^6^ Department for Integrative Evolutionary Biology, Max-Planck Institute for Biology, Max-Planck-Ring 9, 776 Tuebingen, Germany; ^7^ Cell Division Group, Department of Biomedicine, Faculdade de Medicina, Universidade do Porto, Alameda Prof. Hernâni Monteiro, 420-319 Porto, Portugal

**Keywords:** Kinetochore, CENP-E, Kinesin, Chromosome, Holocentric, Mitosis

## Abstract

Chromosome alignment during mitosis can occur as a consequence of bi-orientation or is assisted by the CENP-E (kinesin-7) motor at kinetochores. We previously found that Indian muntjac chromosomes with larger kinetochores bi-orient more efficiently and are biased to align in a CENP-E-independent manner, suggesting that CENP-E dependence for chromosome alignment negatively correlates with kinetochore size. Here, we used targeted phylogenetic profiling of CENP-E in monocentric (localized centromeres) and holocentric (centromeres spanning the entire chromosome length) clades to test this hypothesis at an evolutionary scale. We found that, despite being present in common ancestors, CENP-E was lost more frequently in taxa with holocentric chromosomes, such as Hemiptera and Nematoda. Functional experiments in two nematodes with holocentric chromosomes in which a CENP-E ortholog is absent (*Caenorhabditis elegans*) or present (*Pristionchus pacificus*) revealed that targeted expression of human CENP-E to *C. elegans* kinetochores partially rescued chromosome alignment defects associated with attenuated polar-ejection forces, whereas CENP-E inactivation in *P. pacificus* had no detrimental effects on mitosis and viability. These data showcase the dispensability of CENP-E for mitotic chromosome alignment in species with larger kinetochores.

## Teaser

**Highlighted Article:** Phylogenetic profiling across holocentric or monocentric metazoans, combined with functional experiments in nematodes, supports an inverse relationship between kinetochore size and CENP-E dependence for chromosome alignment.

## INTRODUCTION

At the onset of mitosis, DNA is packed into chromosomes, the nuclear envelope breaks down, and scattered chromosomes start to interact with spindle microtubules via proteinaceous structures that localize on the centromeric region of each sister chromatid, known as kinetochores (Musacchio and Desai, 2017). In vertebrates, when chromosomes are favorably positioned between the two spindle poles, they establish end-on kinetochore–microtubule attachments and align soon after bi-orientation, a pathway known as ‘direct congression’ (Auckland and McAinsh, 2015; Barisic et al., 2014; Klaasen et al., 2022; Maiato et al., 2017). In contrast, more peripheral chromosomes are first brought to the vicinity of spindle poles along laterally attached astral microtubules by the microtubule minus-end-directed motor protein dynein at kinetochores (Li et al., 2007; Vorozhko et al., 2008; Yang et al., 2007). Subsequently, chromosomes are transported towards the equator by the dominant role of the microtubule plus-end-directed kinesin motor CENP-E (kinesin-7), also at kinetochores, that prevents random polar-ejection forces (PEFs) assisted by chromokinesins on chromosome arms (Barisic et al., 2014; Kapoor et al., 2006). Thus, at least in vertebrates, CENP-E at kinetochores is the critical motor driving the alignment of peripheral chromosomes to the cell equator.

One often underappreciated aspect underlying chromosome interactions with mitotic spindle microtubules is kinetochore size. Kinetochore size varies among different animal and plant species (Comings and Okada, 1971; Maddox et al., 2004; Maiato et al., 2006; Malheiros et al., 1947; McEwen et al., 1998; Neumann et al., 2015; Ribeiro et al., 2009), between different chromosomes within a given species (including humans) (Cherry et al., 1989; Cherry and Johnston, 1987; Drpic et al., 2018; Logsdon et al., 2024; McEwen et al., 1998; Nixon et al., 2017; Parl, 2024; Peretti et al., 1986; Sacristan et al., 2024; Sanchez et al., 1991; Tomkiel et al., 1994), and in response to microtubule attachments throughout mitosis (Hoffman et al., 2001; Magidson et al., 2015; Sacristan et al., 2018). How kinetochore size impacts the mechanisms of chromosome alignment remains poorly understood. Based on previous live-cell recordings of Indian muntjac cells with only three large monocentric chromosome pairs and distinct/resolvable kinetochore sizes, we speculated that chromosomes with larger kinetochores depend less on CENP-E activity to bi-orient and align because a larger kinetochore surface area (and possibly a more favorable shape) favors the interaction with spindle microtubules (Drpic et al., 2018). Interestingly, despite the presence of CENP-E in branches of all major eukaryotic lineages suggesting that it was present in the last eukaryotic common ancestor (van Hooff et al., 2017), some metazoans with holocentric chromosomes, such as the *Caenorhabditis elegans* nematode, the kinetochores of which extend along the entire chromosome length, lack a bona fide CENP-E ortholog (Maddox et al., 2004). In this case, proper bi-orientation of sister kinetochores occurs via ‘direct congression’, where kinetochores on holocentric chromosomes initially capture microtubules from fully separated centrosomes soon after nuclear envelope breakdown (NEBD) (Redemann et al., 2017), in a process that is facilitated by the non-kinetochore chromokinesin KLP-19 (Kinesin-4) that assists PEFs required for the stabilization of end-on kinetochore–microtubule attachments (Powers et al., 2004). Whether this represents a *C. elegans* peculiarity or is a more general feature of species with holocentric chromosomes remained unclear. Altogether, these data raise the exciting possibility that CENP-E and the associated, motor-dependent, chromosome alignment pathway became dispensable for mitosis and were lost during evolution in species with relatively large kinetochores, such as those in holocentric chromosomes.

Because absolute kinetochore sizes remain largely unknown for most species, here, we set out to test this hypothesis by systematically investigating the presence of CENP-E orthologs in phylogenetically diverse metazoan taxa with either holocentric or monocentric chromosomes for which high-quality genome sequence data are available. Strikingly, we found a marked propensity for CENP-E loss in taxa with holocentric chromosomes and relatively larger kinetochores compared to taxa with monocentric chromosomes. We then focused our functional analysis on two closely related Nematoda with holocentric chromosomes, in which a CENP-E ortholog was either absent (*C. elegans*) or present (*Pristionchus pacificus*). Remarkably, targeted expression of human CENP-E to *C. elegans* kinetochores did not affect the normal course of mitosis and it was sufficient to partially rescue chromosome alignment defects imposed by the experimental attenuation of PEFs in this system. In turn, CRISPR/Cas9-mediated inactivation of CENP-E in *P. pacificus* had no clear detrimental effects on mitosis during embryogenesis and larval stages, giving rise to fully viable animals. These data strengthen the notion that CENP-E is dispensable even in holocentric species in which it is normally present and provide evidence supporting an inverse relationship between kinetochore size and CENP-E dependence for mitotic chromosome alignment across metazoans.

## RESULTS AND DISCUSSION

### Phylogenetic analysis of CENP-E orthologs in metazoans with monocentric versus holocentric chromosomes

**Fig. 1.**
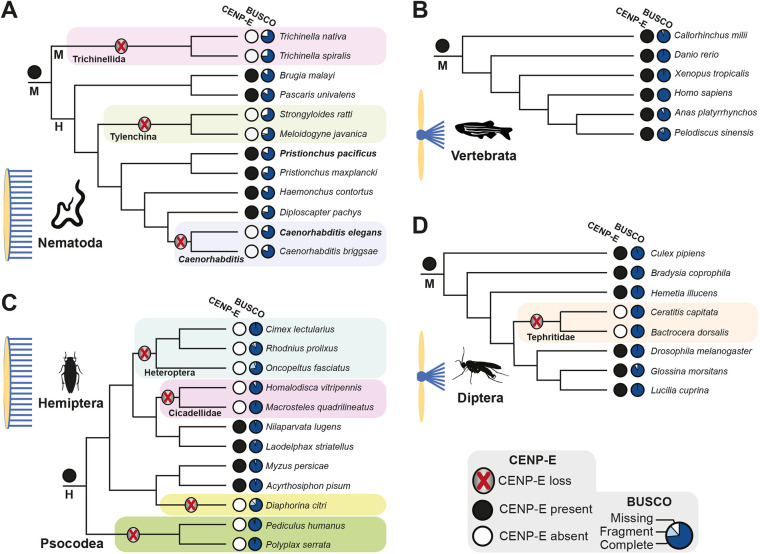
**Phylogenetic profile of CENP-E across holocentric and monocentric taxa.** (A–D) CENP-E conservation in (A) the phylum Nematoda, (B) the phylum Vertebrata, (C) the insect order Hemiptera and (D) the insect order Diptera. Lineages with an inferred CENP-E loss are highlighted with a colored box. Holocentric lineages are indicated with ‘H’ and monocentric lineages with ‘M’, as well as with a graphic depiction of holocentric and monocentric chromosomes. Species silhouettes are from https://www.phylopic.org/, licensed under a CC0 1.0 Universal Public Domain Dedication: Nematoda, *Trichinella sp.*; Vertebrata, *Danio rerio*; Hemiptera, *Nilaparvata lugens*; Diptera, *Bradysia coprophila*. BUSCO scores provide a quantitative assessment of genome assembly and annotation completeness with single-copy orthologs.

To systematically investigate the relationship between kinetochore size and the essentiality of CENP-E in a phylogenetic setting, we profiled the presence or absence of CENP-E in a selection of holocentric and monocentric metazoan taxa through highly sensitive hidden Markov model-based homology searches coupled with phylogenetic analysis. Our species selection comprised taxa at two taxonomic levels of comparable age for which there are high-quality sequence data and for which information on kinetochore type is available (Table S1). We compared the conservation of CENP-E in the predominantly holocentric phylum Nematoda [∼586 million years (Ma) old] to the monocentric phylum Vertebrata (∼462 Ma old). At relatively closer evolutionary timescales and between more similar organisms, we compared CENP-E conservation between the holocentric insect order Hemiptera (true bugs, ∼290 Ma old) and the monocentric insect order Diptera (flies, ∼241 Ma old). We additionally included species from the holocentric order Psocodea (lice, ∼200 Ma old) (Pittendrigh et al., 2006), the sister clade of Hemiptera. This framework allowed us to reconstruct the evolutionary history of CENP-E in these taxa with disparate kinetochore architectures. Our analysis shows that CENP-E is present in member species of all four taxa (Fig. 1A–D; Fig. S1). We found no evidence for horizontal gene transfer in our phylogenetic tree of CENP-E homologs on account of its strong congruency with the consensus phylogeny of our selected species. Therefore, we infer the presence of CENP-E in the last common ancestors of the included taxa, in line with the broad distribution of CENP-E orthologs across eukaryotes (van Hooff et al., 2017). This allowed us to identify subsequent losses along the phylogeny of each of these clades. We found that CENP-E is universally conserved across Vertebrata (Fig. 1B). In contrast, we inferred at least three independent losses of CENP-E in Nematoda (Fig. 1A). Two of these losses were in bona fide holocentric lineages of Nematoda, namely in the order Tylenchina and in the genus *Caenorhabditis*. The third loss was found in the order Trichinellida, which has monocentric chromosomes (Mutafova et al., 1982). Thus, loss of CENP-E appears not to be exclusive to species with holocentric chromosomes. However, we note that this lineage underwent extensive genomic streamlining (wholesale purging of genes) (Mitreva et al., 2011). When comparing the insect orders Hemiptera and Diptera, we found that CENP-E is absent in most Hemiptera lineages, as a result of at least three independent losses in this ancestrally holocentric clade (Fig. 1C,D). Furthermore, we found no CENP-E orthologs in its holocentric sister order Psocodea (Fig. 1C). In comparison, CENP-E is present throughout the monocentric Diptera, with the exception of Tephritidae (peacock flies) (Fig. 1D). Overall, although the type of centromere does not dictate the presence or absence of CENP-E, these analyses show a marked propensity for CENP-E loss in taxa with holocentric chromosomes and relatively larger kinetochores compared to taxa with monocentric chromosomes.

### Targeted expression of human CENP-E to *C. elegans* kinetochores does not compromise mitosis or embryo viability

**Fig. 2.**
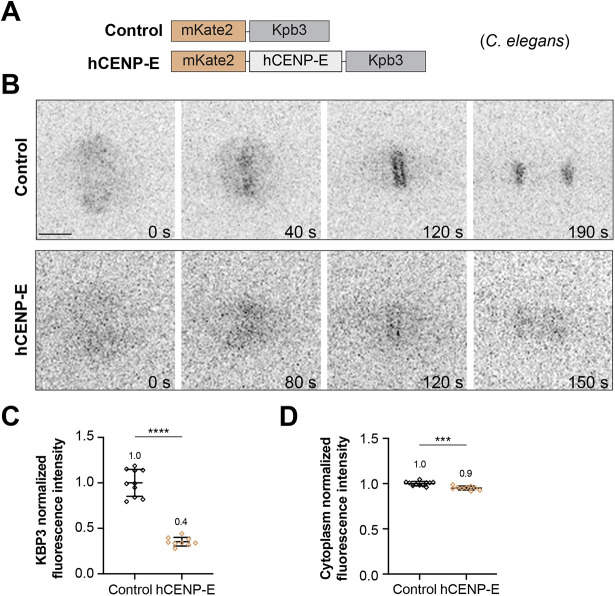
**Expression of human CENP-E in *C. elegans*.** (A) Schematic representation of the transgenes used in this study: KBP-3 (control) and hCENP-E::KBP-3 (hCENP-E) were fused to mKate2 in order to visualize kinetochores. (B) Selected still images from time-lapse sequences of the first embryonic division showing the kinetochore localization of the two constructs (mKate2, inverted grayscale). Scale bar: 5 µm. Timestamps are shown in seconds (s). (C) Normalized fluorescence intensity of mKate2 fusions at kinetochores. (D) Normalized fluorescence intensity of the cytoplasmic signal. Bars show mean±s.d. ***P<0.001; ****P<0.0001; two-tailed unpaired *t*-test. Control: *n*=10 embryos; hCENP-E: *n*=9 embryos.

Given the tendency of CENP-E loss in species with holocentric chromosomes, we next directly investigated the relationship between kinetochore size and CENP-E dependence for chromosome alignment. To this end, we targeted codon-optimized human CENP-E (hCENP-E) expression to *C. elegans* kinetochores that span over 4 µm in length along the entire chromosome in early embryos (Ladouceur et al., 2015; Rocha et al., 2023) and lack a CENP-E ortholog. In vertebrates, CENP-E localizes at the expandable kinetochore fibrous corona throughout mitosis (Cooke et al., 1997; Craske and Welburn, 2020; Yen et al., 1991). Taking this into account, while ensuring the correct kinetochore localization of transgene-encoded mKate2::hCENP-E in *C. elegans* embryos, we fused it to the Spc25 ortholog KBP-3 (Cheeseman et al., 2004). A construct containing mKate2::KBP-3 was used as a control (Fig. 2A). Both constructs were expressed from the germline promoter *Pmex-5*. Kinetochore localization of mKate2-tagged KBP-3 and hCENP-E::KBP-3 during the first embryonic division was confirmed by live-cell imaging (Fig. 2B). Despite codon optimization, the expression levels of mKate2::CENP-E::KBP-3 were reduced to approximately 40% and 95% of mKate2::KBP-3 levels at the kinetochores and cytoplasm, respectively (Fig. 2C,D), perhaps due to protein folding issues or transgene silencing mechanisms operating in the *C. elegans* germline (Aljohani et al., 2020).

**Fig. 3.**
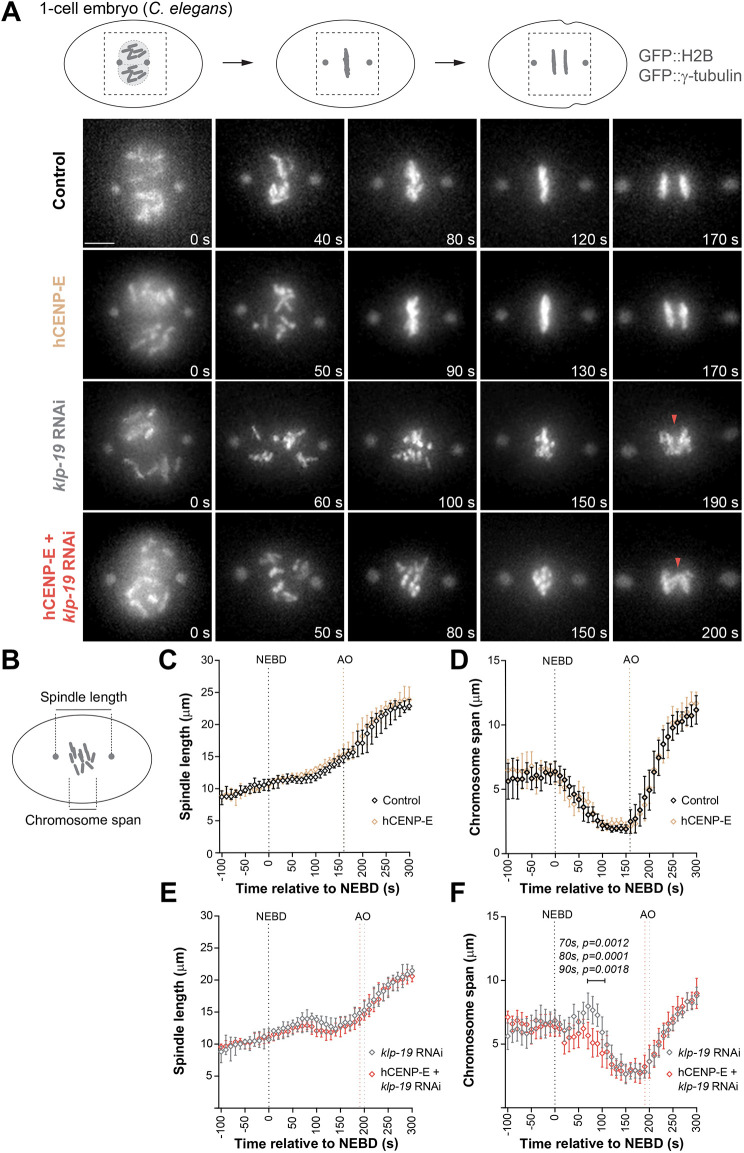
**Expression of hCENP-E does not compromise mitotic progression and rescues chromosome alignment defects due to attenuated polar-ejection forces in *C. elegans*.** (A) Representative live-cell imaging examples of the first embryonic division of control, hCENP-E, *klp-19* RNAi and *klp-19* RNAi+hCENP-E embryos stably expressing GFP::H2B to visualize chromosomes and GFP::γ-tubulin to label the spindle poles. Red arrowheads indicate lagging chromosomes. Scale bar: 5 µm. (B) Schematics of the two quantitative measurements extracted from the live-cell recordings. (C,D) Spindle pole separation (C) and chromosome alignment kinetics (D) in one-cell embryos, showing the same unperturbed effect in the absence (control) and presence of hCENP-E. (E,F) *klp-19* depletion induced a slight premature pole separation at ∼120 s after nuclear envelope breakdown (NEBD; time zero on the plots) (E) and chromosome scattering (F). Repeated measures two-way ANOVA showed no differences in spindle length in embryos expressing hCENP-E [*F*(20, 400)=1.29, *P*=0.1775)], but a significant change in chromosome span [*F*(20, 400)=2.92, *P*<0.0001)]. Post hoc significant comparisons between *klp-19* RNAi and *klp-19* RNAi+hCENP-E are highlighted in the graph: 70 s, *P*=0.0012; 80 s, *P*=0.0001; 90s, *P*=0.0018. Distances were measured in images acquired every 10 s, averaged for the total number of embryos, and plotted against time. Error bars represent the 95% c.i. Black dashed lines represent NEBD. Anaphase onset (AO) for each condition is shown in a dashed line of the corresponding color. *N*=2 independent experiments (control, *n*=13 embryos; control+*klp-19* RNAi, *n*=11 embryos; hCENP-E, *n*=12 embryos; hCENP-E+*klp-19* RNAi, *n*=11 embryos).

**Fig. 4.**
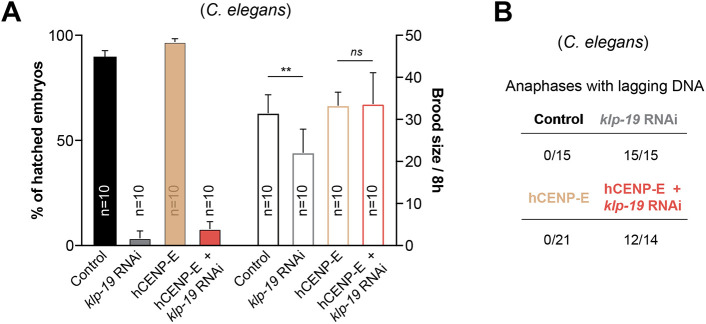
**Expression of hCENP-E rescues brood size to control levels in *C. elegans* with defective polar-ejection forces.** (A) Effect of hCENP-E expression on embryo viability and brood size, with and without *klp-19* RNAi. The mean of *n* mothers ±95% c.i. is shown. ns, not significant; **P<0.01; unpaired two-tailed *t*-test. (B) Frequency of one-cell embryos with anaphases with lagging DNA that persisted for two consecutive frames after anaphase onset; total numbers of analyzed embryos are indicated.

To characterize mitotic progression in the transgenic worms, animals stably expressing mKate2::CENP-E::KBP-3 and mKate2::KBP-3 were crossed with animals expressing GFP::H2B and GFP::γ-tubulin, thus enabling tracking of chromosomes and the spindle poles, respectively. Live-cell imaging of one-cell embryos unveiled that the expression of neither mKate2 fusion constructs perturbed mitosis or compromised embryo viability (Figs 3A and 4A). Two parameters were quantified over time: spindle length, defined as the distance between the two spindle poles; and chromosome span, defined as the maximal distance between the outermost chromosomes along the spindle axis (Fig. 3B). Both parameters remained unaltered in hCENP-E-expressing embryos compared with control embryos (Fig. 3C,D), thus ruling out unspecific effects associated with hCENP-E expression.

### hCENP-E expression partially rescues chromosome alignment defects associated with attenuated PEFs in *C. elegans* embryos

To generate embryos with defective chromosome alignment without directly interfering with end-on kinetochore–microtubule attachments, we attenuated PEFs on chromosome arms by knocking down the *C. elegans* chromokinesin KLP-19 (Powers et al., 2004). As expected, under these conditions, chromosomes became scattered in the cytoplasm after NEBD and took longer to align at the spindle equator, ultimately leading to lagging chromosomes in anaphase (Figs 3A,D,F and 4B). In the first mitotic division, premature spindle pole separation prior to anaphase is indicative of impaired formation of load-bearing kinetochore–microtubule attachments capable of moving chromosomes (Desai et al., 2003; Gassmann et al., 2008; Oegema et al., 2001). As predicted, *klp-19*-depleted embryos displayed premature spindle pole separation starting around 30 s after NEBD. The spindle length decreased around 110 s after NEBD and remained shorter than that in controls at anaphase onset (Fig. 3E). Premature spindle pole separation roughly overlapped with the moment when chromosome span increased, suggesting that chromosomes had not established proper bipolar attachments, with spindle microtubules capable of resisting cortical pulling forces exerted on the poles (Fig. 3F). To test whether hCENP-E could rescue the observed chromosome alignment defects, we imaged one-cell embryos stably expressing mKate2::hCENP-E::KBP-3 after depletion of *klp-19* by RNAi (Fig. 3A). Under these conditions, the delay in establishing load-bearing microtubule attachments was still present (premature pole separation observed 30–40 s after NEBD), suggesting the incapacity of CENP-E to rescue attachment defects. However, the extended chromosome span normally observed after depletion of *klp-19* was partially rescued in hCENP-E-expressing embryos (chromosomes remained closer to the spindle equator) (Fig. 3F). The partial rescue might be explained by the fact that, compared with KBP-3 alone, hCENP-E::KBP-3 expression and the respective kinetochore association in *C. elegans* embryos were significantly lower. Moreover, we found that detyrosinated α-tubulin, which enhances hCENP-E processivity and plays a role in the alignment of pole-proximal chromosomes in human cells (Barisic et al., 2015), was almost exclusively localized to the centrosome region in one-cell *C. elegans* embryos (Fig. S2) and might compromise optimal hCENP-E function in the engineered animals. Of note, although hCENP-E expression did not rescue the viability of *klp-19*-depleted embryos (Fig. 4A), likely owing to enduring lagging chromosomes during anaphase (Fig. 4B), it rescued the brood size to control levels (Fig. 4A). These data provide *in vivo* evidence that hCENP-E is functional when expressed in *C. elegans* embryos and is able to overcome defects associated with other congression pathways that would normally benefit from having holocentric chromosomes with larger kinetochores. Moreover, they open the exciting possibility that species with holocentric chromosome architecture have lost CENP-E due to its dispensability for chromosome alignment and mitosis in general.

### CENP-E inactivation in *P. pacificus* does not compromise mitosis and viability

In contrast to *C. elegans*, the *P. pacificus* genome contains a CENP-E ortholog that is annotated in the current version of the genome as PPA38637 (ElPaco at http://pristionchus.org/). We named this gene *Ppa-cenp-E* following standard nematode nomenclature rules. We generated three independent alleles (*tu1915*, *tu1916* and *tu1917*) with a 4 bp deletion and 20 bp and 13 bp insertions, respectively, all of which resulting in frameshift mutations. All three mutant lines were viable and grew normally with no obvious difference in lifespan and/or accumulating lethality over generations. These data indicate that CENP-E is dispensable for mitosis even in holocentric species in which it is normally present, but the kinetochores of which are one order of magnitude larger (up to 2 µm in length) than in most monocentric species (Rillo-Bohn et al., 2021). However, we note that brood size counts revealed a higher variability than what is typically observed in *P. pacificus* wild-type PS312 animals (normally between 120 and 180 progeny per hermaphrodite). Specifically, we found 55–213 (*tu1915*), 75–269 (*tu1916*) and 93–217 (*tu1917*) progeny in the three mutant lines (Table S2). These numbers suggest that, although dispensable for mitosis, CENP-E has been retained in *P. pacificus* due to other roles, such as the ‘sperm–oocyte switch’ that determines progeny number and might confer a fitness advantage.

### Study limitations and outlook

We recognize that our study has limitations in breadth, imposed mostly by the lack of information on absolute kinetochore size in the majority of the species analyzed. Nevertheless, we contend that comparing species with holocentric chromosomes in which the kinetochore extends along the entire chromosome length with species with localized kinetochores that normally cannot be resolved by light microscopy (i.e. they are less than 200 nm in length) is a reasonable first approximation to the problem, even taking into consideration that chromosome (and kinetochore) size scales with a reduction in cell and nuclear size during embryonic development (Ladouceur et al., 2015). For instance, kinetochore sizes in the two nematode species used in the present study for experimental follow-up after phylogenetic analyses correspond to 4 µm and 2 µm in *C. elegans* (Rocha et al., 2023) and *P. pacificus* early embryos (Rillo-Bohn et al., 2021), respectively. Therefore, the tendency to lose CENP-E during eukaryotic evolution appears to correlate with a larger kinetochore size, and this is particularly evident among species with holocentric chromosomes. For instance, *C. elegans* embryos rapidly bi-orient and align all chromosomes within approximately 120 s after NEBD, and only ∼1% of anaphases show lagging chromosomes (Stear and Roth, 2002). However, when experimentally challenged, *C. elegans* embryos can have high rates of chromosome alignment defects and missegregation events (Cheeseman et al., 2005; Desai et al., 2003; Gassmann et al., 2008; Oegema et al., 2001; Powers et al., 2004; Stear and Roth, 2002). Remarkably, *C. elegans* engineered to express hCENP-E fused to the kinetochore protein KBP-3 did not show any problems throughout mitosis or in animal viability, whereas the related holocentric worm *P. pacificus*, which harbors a bona fide CENP-E ortholog, was able to survive and grow normally without CENP-E. These experiments provide compelling evidence that CENP-E is dispensable for chromosome alignment during mitosis in well-established model systems with holocentric chromosomes, possibly due to a compensatory mechanism associated with their larger kinetochore size. Nevertheless, CENP-E appears to be important for the sperm–oocyte switch in *P. pacificus*, possibly reflecting recently uncovered roles of CENP-E during spermatogenesis (She et al., 2021, 2020; Zhang et al., 2024).

## MATERIALS AND METHODS

### Proteome selection

We assembled a dataset of predicted proteomes from holocentric and monocentric species. The species were selected to span the known diversity of the holocentric taxa Nematoda and Hemiptera, and the monocentric taxa Vertebrata and Diptera. The divergence times of these taxa were estimated with https://timetree.org/ (last accessed 19 June 2024) (Kumar et al., 2017). To measure the quality of the predicted proteomes prior to inclusion for this study, we performed a BUSCO (v5.7.1) completeness analysis using the metazoan_odb10 dataset (Manni et al., 2021). The final dataset comprised 12 Nematoda species, six Vertebrata species, ten Hemiptera species, two Psocodea species and eight Diptera species, totaling 38 species. We filtered out isoforms of the human proteome by annotation in the original sequence identifiers. The sources of the used proteomes and BUSCO completeness scores are listed in Table S1.

### Homology detection

To find homologs of CENP-E in each of the selected species, we ran the hmmsearch method from the HMMER3 package (v3.1b2; http://hmmer.org/) with all heuristic filters turned off for maximum sensitivity (--max option). Specifically, we queried the CENP-E motor domain-specific hidden Markov model from Tromer et al. (2019) against each predicted proteome in our dataset. The hits were sorted on ascending domain e-value, and hits with identical domain e-values were filtered to remove redundant hits. Then, the best 20 hits were selected for further phylogenetic analysis.

### Phylogenetic analysis

Delineating orthologs from a set of homologs requires robust phylogenetic analysis. Here, we took the best 20 homology hits from each of the selected proteomes and these were collated. Then, a multiple sequence alignment (MSA) was created from the homologs using MAFFT E-INS-i (Katoh and Standley, 2013). The resulting MSA was filtered manually by excluding poorly aligned sequences and truncated to exclusively contain the conserved motor domain. Additionally, the alignment was trimmed to remove positions with ≤70% occupancy using trimAl (Capella-Gutiérrez et al., 2009). A phylogenetic tree was inferred from the MSA with IQ-TREE using ModelFinder to find the best-fitting substitution model (Kalyaanamoorthy et al., 2017; Nguyen et al., 2015). IQ-TREE was run ten independent times to maximize the likelihood of the topology inference, in accordance with Zhou et al. (2018), and UFBoot2 was used to get bootstrap support for branches with 1000 replicates (Hoang et al., 2018). The inferred phylogeny was visualized in iTOL (Letunic and Bork, 2021). To annotate the orthologous clades in the phylogeny, a blastp search was conducted for each of the hit sequences against the Swiss-Prot database (Altschul et al., 1990; Sayers et al., 2022). An interactive version of our phylogenetic tree is available via https://itol.embl.de/tree/145909325331361721030804, hosted by the iTOL website.

### Codon bias optimization

In order to increase the probability of human gene expression in *C. elegans*, we performed a codon bias optimization of the human CENP-E sequence (isolated from the 883-CENP-E 620 plasmid, a gift from Michael Lampson, University of Pennsylvania, Philadelphia, USA; Zhang et al., 2017) using the GenScript OptimumGene service. Accordantly, the codon adaptation index and the frequency of optimal codons were calculated to enhance gene expression in this organism. The optimized CENP-E sequence used for this study is given in Table S5 (optimized sequence length: 1906; GC content: 36.70%). The sequence was cloned into a standard cloning pUC57 vector (GenScript) flanked by two EcoRV (GATATC) cutting sites.

### Molecular cloning

The Pmex5_mKate2_KBP-3 and Pmex5_mKate2_CENP-E_KBP-3 plasmids were generated in this study from genomic DNA isolated from *C. elegans*, pCFJ151 containing mKate2 (Frokjaer-Jensen et al., 2008) and pUC57-CENP-E (codon optimized). Gibson assembly was used to assemble the two or three DNA fragments, respectively (Gibson, 2009). The primers used for PCR amplification and Gibson assembly are described in Table S3. Gibson Assembly Master Mix (New England Biolabs) was added to the DNA fragments and incubated at 50°C for 1 h. After transforming Top10 competent bacteria with 4–6 µl of the Gibson assembly reaction products, the bacteria were plated in Luria–Bertani (LB) agar plates with ampicillin (ForMedium, AMP25). The following day, colonies were picked to perform a colony PCR. Positive colonies were selected and DNA was extracted using the NZYMiniprep kit (NZYtech). The Sanger sequencing reaction was performed by GenCore (i3S) according to the manufacturer's instructions with the following components: BigDye Terminator v3.1 Cycle Sequencing Kit (Applied Biosystems), BigDye Terminator v1.1 (Thermo Fisher), v3.1 5× Sequencing Buffer (Applied Biosystems), primer (10 µM), nuclease-free water (Ambion) and plasmid (∼100 ng). The results were then analyzed using SnapGene.

### Worm strains

A Mos1 transposon-based strategy (MosSCI) was used to generate a strain stably expressing mKate2::KBP-3 and mKate2::CENP-E::KBP-3 under the control of the *mex-5* promotor and *tbb-2* 3′ untranslated region for expression in germline cells (Frokjaer-Jensen et al., 2012, 2008). Other fluorescent markers were subsequently introduced by mating. The GCP826 (mKate2::KBP-3×GFP::H2B/GFP::TBG-1) and GCP815 (mKate2::CENP-E::KBP-3×GFP::H2B/GFP::TBG-1) strains created in this study were maintained at 20°C on standard nematode growth medium (NGM) plates seeded with *Escherichia coli* OP50 bacteria.

### RNA interference

Double-stranded RNAs for *klp-19* (gene ID: Y43F4B.6) were obtained using the following oligonucleotides – T3 promoter, 5ʹ-aattaaccctcactaaaggTGACCCAGAAGAACTCTCGC-3ʹ; T7 promoter, 5ʹ-taatacgactcactataggTCGGAGATCTTCACACAGCC-3ʹ – and were delivered by injecting L4 hermaphrodites. After injection, animals were incubated for 48 h at 20°C for a penetrant depletion before embryos were isolated for live-cell imaging.

### Live-cell imaging of embryos

Adult gravid hermaphrodite worms were dissected in a watch glass filled with Egg Salts medium (118 mM KCl, 3.4 mM MgCl_2_, 3.4 mM CaCl_2_ and 5 mM HEPES pH 7.4), and embryos were mounted on a fresh 2% agarose pad and covered with an 18 mm×18 mm coverslip (no. 1.5H, Marienfeld). Embryos co-expressing GFP::H2B, GFP::γ-tubulin and mKate2::KBP-3 or mKate2::CENP-E::KBP-3 for tracking of nuclei, centrosomes and kinetochores were imaged on an Axio Observer microscope (Zeiss) equipped with an Orca Flash 4.0 camera (Hamamatsu), a Colibri.2 light source, and controlled by ZEN software (Zeiss). All other imaging was performed on a Nikon Eclipse Ti microscope coupled to an Andor Revolution XD spinning disk confocal system composed of an iXon Ultra 897 CCD camera (Andor Technology), a solid-state laser combiner (ALC-UVP 350i, Andor Technology) and a CSU-X1 confocal scanner (Yokogawa Electric Corporation), controlled by Andor IQ3 software (Andor Technology). All imaging was performed in temperature-controlled rooms kept at 20°C. Time-lapse sequences were processed and analyzed with Fiji software (ImageJ version 2.0.0-rc-56/1.51h).

### Pole-to-pole distance and chromosome span measurements

Embryos expressing GFP::H2B, GFP::γ-tubulin and mKate2::KBP-3 or mKate2::CENP-E::KBP-3 were imaged at 10 s intervals, with nine *z*-slices spaced 1.5 μm apart for the fluorescence channel, and a single central slice per time point for the differential interference contrast (DIC) channel, at 2×2 binning with a 63× NA 1.4 oil immersion objective (Zeiss), from just prior to NEBD in the one-cell embryo until the onset of cytokinesis. Embryo length was defined as the distance between the outermost points of the eggshell visible in the DIC image. After maximum intensity projection of GFP *z*-stacks, the *x* and *y* coordinates of the centrosomes and of the chromosomes closer to each centrosome were recorded over time using the MtrackJ plugin in ImageJ by manually clicking on the center of centrosomes and on the outer edge of the chromosomes.

### Immunofluorescence of *C. elegans* embryos

Ten to 12 adult worms were dissected into 3 μl of M9 buffer (86 mM NaCl, 42 mM Na_2_HPO_4_, 22 mM KH_2_PO_4_ and 1 mM MgSO_4_) on a poly-*L*-lysine-coated slide. A 18×24 mm coverslip was placed on the 3 μl drop, and slides were plunged into liquid nitrogen. After rapid removal of the coverslip (‘freeze-cracking’), embryos were fixed in −20°C methanol for 20 min. Embryos were re-hydrated two times for 5 min in PBS (137 mM NaCl, 2.7 mM KCl, 8.1 mM Na_2_HPO_4_ and 1.47 mM KH_2_PO_4_), blocked with AbDil (PBS with 2% BSA and 0.1% Triton X-100) in a humid chamber at room temperature for 30 min, and incubated with a rabbit monoclonal anti-detyrosinated α-tubulin (1:1000; a gift from Marin Barisic, Danish Cancer Institute, Copenhagen, Denmark; Liao et al., 2019) for 2 h at room temperature. After washing four times for 5 min in PBS, embryos were incubated with Alexa Fluor 488 goat anti-rabbit IgG (1:1000; Thermo Fisher Scientific, A-11029; RRID: AB_143165) for 1 h at room temperature. Embryos were washed four times for 5 min in PBS and mounted in Prolong Gold with DAPI stain (Invitrogen, P36931). Images were recorded on an AxioImager Z1 (100× Plan-Apochromatic oil DIC objective lens, 1.46 NA, Carl Zeiss Microimaging) equipped with a CCD camera (ORCA-R2, Hamamatsu) operated by Zen software (Carl Zeiss). Image files were imported into Fiji for further processing.

### Embryonic viability and brood size counts

Brood size and embryonic viability assays were performed at 20°C. L4 hermaphrodites, injected with *klp-19* RNAi or untreated, were grown for 40 h on NGM plates containing OP50 bacteria. Single adults were then placed on new plates with a small amount of OP50, removed 8 h later, and the plates were incubated for another 16–24 h to give viable embryos time to hatch. Embryonic viability was calculated by dividing the number of hatched larvae by the total number of progenies on the plate. Replicate counts were averaged and plotted as the mean percentage ±95% confidence interval (c.i.).

### Maintenance of *P. pacificus* cultures and brood size counts

CRISPR experiments were done with the *P. pacificus* wild-type strain PS312. Three alleles of *Ppa-cenp-E* were obtained. All worm cultures were propagated at room temperature (20°C) on NGM in 6 cm Petri dishes, as outlined before (Werner et al., 2017). In all experiments, *E. coli* OP50 was used as food source. Bacteria were grown overnight at 37°C in LB medium, and 400 μl of the overnight culture was pipetted on NGM agar plates and left for several days at room temperature to grow bacterial lawns. For brood size counts, single J4 juvenile worms were passed on these lawns and transferred daily over a 5-day period.

### CRISPR/Cas9 mutagenesis in *P. pacificus*

The procedure for CRISPR/Cas9 mutagenesis was based on previously published protocols for *P. pacificus* (Han et al., 2020; Witte et al., 2015). A target-specific CRISPR RNA (crRNA) of the sequence 5′-GCCTACGGACAGTCTGGCAG-3′ was synthesized to target 20 bp upstream of the protospacer adjacent motifs and fused to tracrRNA (1072534; Integrated DNA Technologies) at 95°C for 5 min, and subsequently allowed to cool down to room temperature and anneal. The hybridization product was combined with Cas9 protein (1081058; Integrated DNA Technologies). After a further 5 min incubation at room temperature, TE buffer was added for final concentration of 18.1 µM for the single guide RNA and 12.5 µM for Cas9. A plasmid carrying the *Ppa-eft-3* promoter and modified TurboRFP sequences was used as a co-injection marker (Han et al., 2020). After 2 days, P0 plates containing the F1 animals with fluorescence signal of the co-injection marker were isolated, and eight to ten F1 progenies from these plates were singled out on individual plates. The genotypes of the F1 animals were subsequently analyzed via Sanger sequencing after PCR amplification of a *Ppa-cenp-E* fragment covering the targeted site using the forward primer (5′-GAAGCCTCTCGTTGATGGAACACTTCAGG-3′) and the reverse primer (5′-CACATATTGACATTCTTAGGGCGAAAGTC-3ʹ). Mutations were identified before isolation of homozygous mutant carriers. Detailed information of mutants generated in this study can be found in Table S2.

### Statistical analysis

Statistical analysis was performed with GraphPad Prism 8.1.2 software. D'Agostinho–Pearson omnibus normality test was used to determine whether the data followed a normal distribution. If α=0.05, the difference between the population distributions was determined by two-tailed unpaired Student's *t*-test. Two-tailed unpaired *t*-test was used to compare the fluorescence intensity of KBP-3 at the kinetochores and at the cytoplasm in the transgenic worms, and to analyze the brood size, with and without *klp-19* RNAi. Statistical differences were considered significant at *P*<0.05. To assess the overall effects of hCENP-E expression on *klp-19*-depleted embryos, we performed a repeated measures two-way ANOVA (from 0 to 200 s, NEBD to anaphase onset), followed by post hoc comparisons using Bonferroni correction to evaluate specific time points relevant for chromosome alignment (Table S4). All plots represent mean values and s.d. or 95% c.i. calculated for all replicates. Sample sizes were not predetermined but were obtained based on current best practice in the field.

## Supplementary Material



10.1242/joces.263466_sup1 Supplementary information

Table S1. Information on the species and source of the proteomes used in this study for the phylogenetic analysis.
