# Supplementary material for: An evolutionary perspective on the relationship between kinetochore size and CENP-E dependence for chromosome alignment
Source: J Cell Sci. 2024 Dec 19;137(24):jcs263466. doi: 10.1242/jcs.263466 (PMC11827601; doi:10.1242/jcs.263466)
Supplement: Supplementary information [file joces-137-263466-s1.pdf]

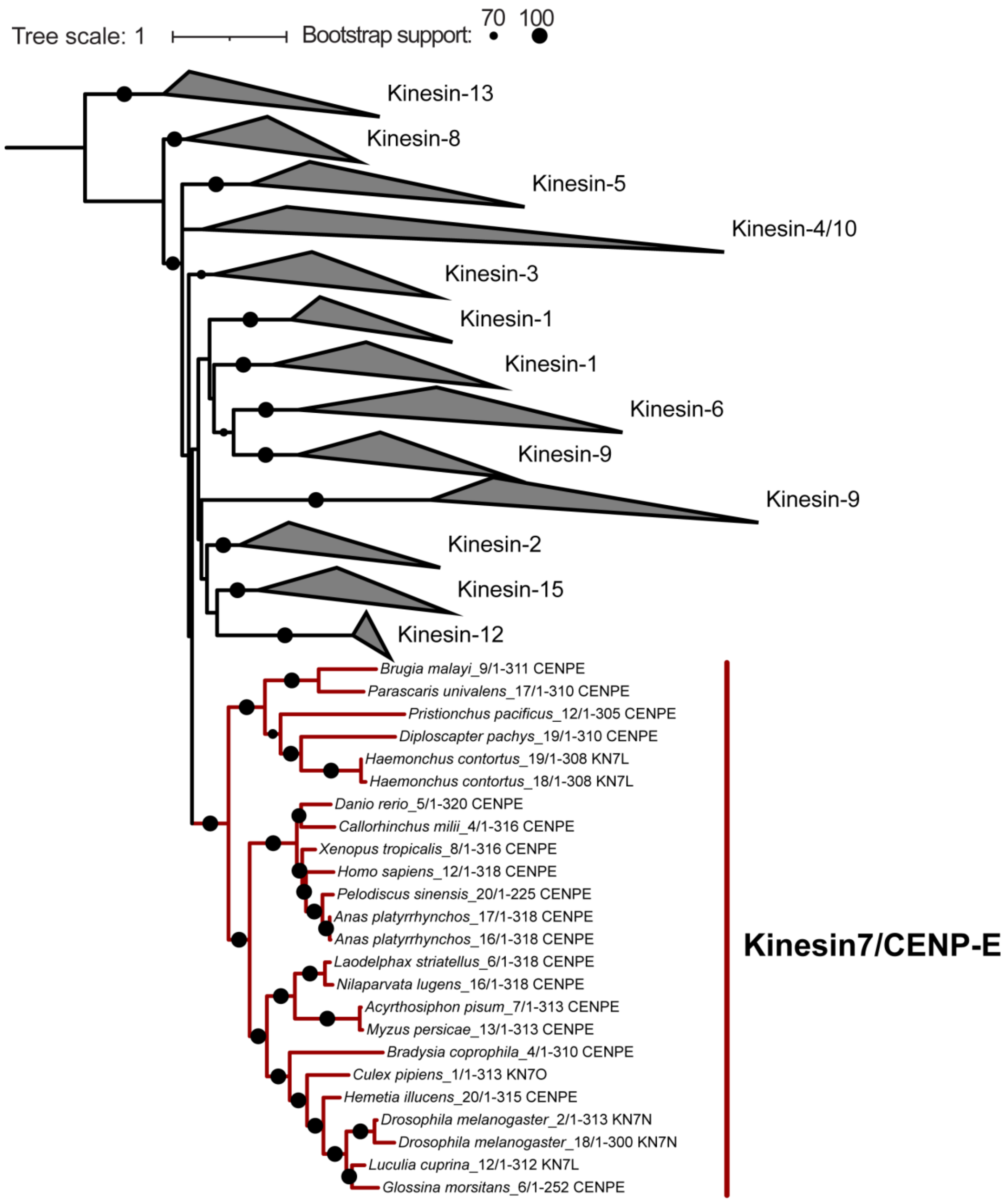

Fig. S1. Phylogenetic tree of 562 CENP-E homologues arbitrarily rooted on the Kinesin-13 clade. The CENP-E orthologous clade is highlighted in red. Outgroup kinesin clades are collapsed. Species names are abbreviated as specified in Supplementary Table S1. The tree was run under the LG+C60+F+R4 substitution model and the tree with the best likelihood was selected out of a set of 10 independent runs. Note: *P. maxplancki* CENP-E does not have a complete motor domain and is therefore not included in the phylogenetic analysis.

Journal of Cell Science • Supplementary information

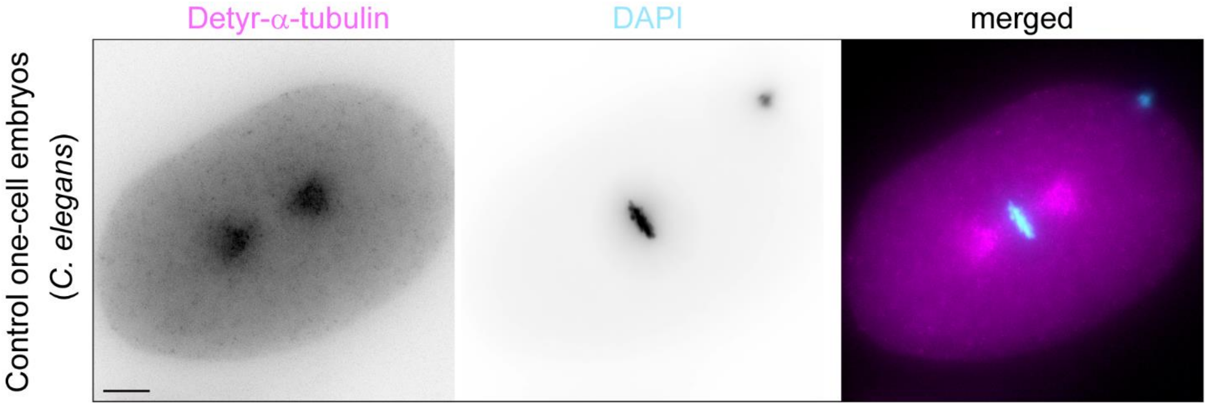

Fig. S2. Detyrosinated microtubules are almost exclusively localized to the centrosome region in one-cell *C. elegans* embryos. Image shows a maximum intensity projection of a representative control one-cell embryo processed for immunofluorescence to visualize detyrosinated-α-tubulin (magenta) and counterstained with DAPI to visualize nuclei (cyan). Scale bar: 5 µm.

Table S1. Information on the species and sources of the proteomes used in this study for the phylogenetic analysis.

Available for download at
https://journals.biologists.com/jcs/article-lookup/doi/10.1242/jcs.263466#supplementary-data

Table S2. *Ppa-cenp-E* alleles.

Allele	Molecular lesion	Targeted exon	Type of error
tu1915	4 bp Deletion	4	Frameshift
tu1916	20 bp Insertion	4	Frameshift
tu1917	13 bp Insertion	4	Frameshift

Journal of Cell Science • Supplementary information

Table S3. Primers used to assemble the plasmids generated in this study.

Template	Primer Fw	Primer Rv	Product size (bp)
**Plasmid pRG777-hCENP-E (backbone)**	AACATCTTGGTCTAAatgcaagatcctttcaagcattcccttcttctctatcac	CGCCCCCTCTTCCGCCATgcttccaccagatccACGGTG	9138
**Genomic DNA from C. elegans**	AAGACCCGGGATCTGGTGGAAGCATGGAGTCACTCAACGAGTATATGGACAAGATTATTAATCGTC	gaaaggatcttgcatTTAGACCAAGATGTTGGAGAGGTTCGATctgaaaaaaatacac	806
**pRG777-kbp-3 (backbone)**	AACATCTTGGTCTAAatgcaagatcctttcaagcattcccttcttctctatcac	GTTGAGTGACTCCATgcttccaccagatccACGGTG	9135
**Genomic DNA from C. elegans (mKate2-kbp-3)**	ggatctggtggaagcATGGAGTCACTCAACGAGTATATGGACAAGATTATTAATCGTC	gaaaggatcttgcatTTAGACCAAGATGTTGGAGAGGTTCGATctgaaaaaaatacac	798

Journal of Cell Science • Supplementary information

Table S4. Results of Repeated Measures Two-Way ANOVA for Spindle Length and Chromosome Span in *klp-19* RNAi and *klp-19* RNAi + hCENP-E *C. elegans* embryos.

**Spindle length**	**Sum of Squares (SS)**	**Degrees of freedom (df)**	**Mean of Squares (SS)**	**F -value**	**P-value**
Time relative to NEBD **x** *klp-19* RNAi +/- hCENP-E	16.01	20	0.80	1.29	P=0.1775
Time relative to NEBD	463.3	20	23.17	37.49	P<0.0001
klp-19 RNAi +/- hCENP-E	37.60	1	37.60	2.15	P=0.1577
Residual (error)	247.2	400	0.62		
**Chromosome Span**	**Sum of Squares (SS)**	**Degrees of freedom (df)**	**Mean of Squares (SS)**	**F -value**	**P-value**
Time relative to NEBD x *klp-19* RNAi +/- hCENP-E	80.60	20	4.03	2.92	P<0.0001
Time relative to NEBD	1140	20	57.00	41.25	P<0.0001
klp-19 RNAi +/- hCENP-E	71.09	1	71.09	7.77	P=0.0114
Residual (error)	552.8	400	1.38		

Journal of Cell Science • Supplementary information

Table S5. Optimized CENP-E sequence used for this study.

GGAGCCGTCGCCGTCTGCGTTCGTGTCCGTCCATTGAATAGTCGTGAAGAAAGTCTGGGAGAAACAGC
ACAAGTCTACTGGAAAACAGATAACAACGTTATCTACCAAGTTGATGGATCTAAATCATTCAACTTCGAT
AGAGTTTTCCATGGAAACGAAACAACTAAGAACGTTTACGAAGAAATTGCTGCACCAATCATCGATTCT
GCTATCCAAGGATACAACGGAACTATCTTTGCTTACGGACAAACAGCATCTGGAAAAACATATACTATGA
TGGGATCAGAAGATCATTTGGGAGTTATTCCACGAGCAATCCATGATATTTTCCAAAAGATTAAGAAATT
CCCAGATAGAGAATTCCTTTTGCGAGTTTCATACATGGAAATCTACAACGAAACAATTACTGATCTTTTG
TGTGGAACACAAAAGATGAAGCCACTTATCATCAGAGAAGATGTTAACCGAAACGTTTACGTTGCTGAT
TTGACTGAAGAAGTTGTTTACACATCTGAAATGGCACTTAAGTGGATCACTAAGGGAGAAAAGTCAAG
ACATTACGGAGAAACAAAGATGAACCAAAGATCATCTCGATCTCATACTATCTTCAGAATGATCTTGGAA
TCTCGAGAAAAAGGAGAACCATCAAATTGTGAAGGATCTGTTAAAGTTTCACATCTTAACTTGGTTGAT
TTGGCTGGATCAGAACGAGCTGCACAAACTGGAGCTGCAGGAGTTAGACTTAAGGAAGGATGCAACAT
CAACCGATCTCTTTTCATCTTGGGACAAGTTATTAAGAAACTTTCAGATGGACAAGTTGGAGGATTCATC
AACTACAGAGATTCTAAGCTTACACGAATCTTGCAAAACTCACTTGGAGGAAACGCTAAGACTAGAATC
ATCTGCACAATCACTCCAGTTTCTTTCGATGAAACACTTACTGCATTGCAATTCGCTTCAACTGCAAAGT
ACATGAAGAACACACCATACGTTAACGAAGTTTCTACAGATGAAGCTCTTTTGAAAAGATACCGAAAGG
AAATCATGGATTTGAAAAAACAACTTGAAGAAGTTTCATTGGAAACTAGAGCTCAAGCAATGGAAAAA
GATCAACTTGCACAACTTTTGGAAGAAAAGGATCTTTTGCAAAAGGTTCAAAACGAAAAGATCGAAAA
CTTGACAAGAATGCTTGTTACTTCATCTTCACTTACATTGCAACAAGAATTGAAAGCTAAACGAAAAAG
ACGAGTTACATGGTGTCTTGGAAAGATCAACAAGATGAAGAACTCAAACTATGCAGATCAATTCAATAT
TCCAACTAATATTACAACTAAAACACATAAATTGTCTATTAATCTTTTGAGAGAAATTGATGAATCAGTTT
GCTCTGAATCAGATGTTTTCTCAAACACACTTGATACTTTGTCAGAAATCGAATGGAATCCAGCTACTAA
GCTTTTGAACCAAGAAAACATCGAATCTGAACTTAACTCATTGAGAGCAGATTACGATAACCTTGTTTTG
GATTACGAACAATTGCGAACAGAAAAGGAAGAAATGGAACTTAAGTTGAAGGAAAAGAATGATTTGGA
TGAATTCGAAGCTCTTGAAAGAAAGACTAAAAAAGATCAAGAAATGCAACTTATCCATGAAATTTCAAA
CCTTAAGAACTTGGTTAAGCATGCAGAAGTTTACAACCAAGATTTGGAAAACGAACTTTCTTCAAAGGT
TGAACTTTTGCGAGAAAAGGAAGATCAAATTAAGAAACTTCAAGAATACATCGATAGTCAAAAACTGG
AGAACATCAAAATGGATCTGTCCTACTCGCTGGAA

Journal of Cell Science • Supplementary information
