# Supplementary material for: An evolutionary perspective on the relationship between kinetochore size and CENP-E dependence for chromosome alignment
Source: J Cell Sci. 2024 Dec 19;137(24):jcs263466. doi: 10.1242/jcs.263466 (PMC11827601; doi:10.1242/jcs.263466)
Supplement: Supplementary file 1 [file joces-137-263466_review_history.pdf]

An evolutionary perspective on the relationship between kinetochore size and CENP-E dependence for chromosome alignment

Ana C. Almeida, Helder Rocha, Maximilian W. D. Raas, Hanh Witte, Ralf J. Sommer, Berend Snel, Geert J. P. L. Kops, Reto Gassmann, Helder Maiato
DOI: 10.1242/jcs.263466

Editor: Renata Basto

Review timeline
Original submission: 2 August 2024
Editorial decision: 13 September 2024
First revision received: 24 October 2024
Accepted: 13 November 2024

Original submission

First decision letter

MS ID#: JOCES/2024/263466

MS TITLE: An evolutionary perspective on the relationship between kinetochore size and CENP-E dependence for chromosome alignment

AUTHORS: Ana C. Almeida; Helder Rocha; Max W. D. Raas; Hanh Witte; Ralf J. Sommer; Berend Snel; Geert J. P. L. Kops; Reto Gassmann; Helder Maiato
ARTICLE TYPE: Short Report

We have now reached a decision on the above manuscript.

As you will see, one of the reviewers raise a number of substantial criticisms that prevent us from considering the paper at this stage. If you think that you can deal satisfactorily with the criticisms on revision, we would be pleased to see a revised manuscript. We would then return it to the reviewers.

Reviewer 1

Advance summary and potential significance to field

Almeida et al. report on the relationship between kinetochore size and CENP-E dependence for chromosome alignment in different taxa. Using targeted phylogenetic profiling on CENP-E under mono- versus holocentric conditions, the authors report that this protein was more frequently lost in taxa with holocentric chromosomes. Briefly, analysis in two nematodes revealed that expression of human CENP-E in C. elegans (the CENP-E orthologue is absent in the species) partially rescued chromosome alignment, while CENP-E inactivation in P. pacificus (the CENP-E orthologue is present in the species) had no effect on mitosis.

This paper reports on an interesting aspect of kinetochore biology and the format of a report in the JCS is appropriate for this message to the cell biology community. I have no major points of

© 2024. Published by The Company of Biologists under the terms of the Creative Commons Attribution License (https://creativecommons.org/licenses/by/4.0/).

criticism but I would like the authors to take care of some issues (see below) to improve the presentation of the data.

Comments for the author

Figures 3 and 4 should be rearranged! The information here is kind of splitted. A jumping from figure 3 to 4 and vice versa is necessary to understand the message. This needs to be avoided. Fig. 3A and Fig. 4A should be combined and shown in one panel. The schematic drawings should be inserted into this new Fig. 3A. In addition, Figure 3C and Figure 4B, and also Figure 3D and Figure 4C should be fused. This will be new Figure 3 B and C. Last, Figure 4D and E would be the new Figure 4A and B.

Please make sure to explicitly mention the nematode species (C. elegans or P. pacificus) in the figure legends. This should always be very clear.

I'm not sure about the whole last paragraph of the results section. I realize that a lot of work went into this aspect. In the end, however, what does it really add to this paper. The statement about an effect on the sperm-oocyte switch is very, very general.

Supplementary Figure 1 needs some explanation and extended information. I think this part of the experiments is not properly explained in the experimental procedures.

Additional small issue:
Line 77: change "works" to "work".

<u>Reviewer 2</u>

Advance summary and potential significance to field

The authors of this study follow up on previous work showing that chromosomes with larger kinetochores (in Indian muntjac deer) tend to bi-orient at the metaphase plate without using the motor CENP-E/kinesin-7. Specifically, the authors examine the pattern of CENP-E loss in different animal clades and correlate this with the use of holocentric or monocentric chromosomes. They then carry out some experiments in two nematodes with holocentric chromosomes, one carrying a CENP-E ortholog and one with a CENP-E loss. While the premise is intriguing and the combination of targeted phylogenetic analysis and experiment holds great promise as a general approach to investigate such questions, the study fails to deliver in some critical aspects.

Comments for the author

First, the paper (including the title and abstract) claims to investigate the relationship between kinetochore size and CENP-E dependence. However, the authors never measure kinetochore size, neither the variance in average size between species or the variance within species between the different chromosomes. The only comparison is between a small number of holocentric lineages, which exhibit more frequent CENP-E losses, and monocentric lineages, which exhibit fewer losses. While the trend is interesting, the correlation is hardly striking.

Second, the evolutionary logic presented in this manuscript is not clear. A previous study (with an overlapping author list), Van Hoof et al. EMBO Reports 2017, clearly demonstrates that CENP-E is pan-eukaryotic (with a LECA origin) and in fact greatly expanded through gene duplications in many lineages. Yet on line 91 the authors write: "Altogether, these data raise the exciting possibility that CENP-E and associated, motor-dependent, chromosome alignment pathway, have emerged during evolution to compensate for a reduction in kinetochore size typically observed in vertebrates."

Third, why is the phylogenetic analysis restricted to CENP-E? Surely it would be worth looking at other components of the "associated, motor-dependent, chromosome alignment pathway"? Are these molecular players known? How many are shared with the motor-independent pathway?

© 2024. Published by The Company of Biologists under the terms of the Creative Commons Attribution License (https://creativecommons.org/licenses/by/4.0/).

Fourth, the experiments in the two nematode species do not really report in any way on the relationship between kinetochore size and CENP-E function. It is difficult to conclude anything from the lack of phenotype upon expressing (human) CENP-E in C. elegans - perhaps it is not capable of assuming its normal function within this foreign cellular milieu even if forcibly targeted to kinetochores. (More generally, not much can be confidently inferred from a negative result downstream of in trans expression.) The effect size of the rescue in embryos with attenuated PEF is very small.

Finally, the lack of mitotic phenotypes upon CENP-E inactivation in P. pacificus only shows that there is no obligate dependency relationship in a holocentric species that carries CENP-E - but this would not be expected in any case, especially not given the authors' previous work - as they state in Drpic et al. Current Biology 2018, "Any Chromosome May Use Either the CENP-E-Dependent or -Independent Pathway to Congress, Regardless of Kinetochore Size".

Thus, overall, I find that the major conclusions of the study, and in particular its central premise, are not really supported by the analyses and experiments presented here, and would urge the authors to include a much more comprehensive phylogenetic analysis supported by experiments that actually measure kinetochore sizes in species selected for experimental follow-up.

Minor:

It is not appropriate to apply t-tests to individual timepoints from continuous time course data (Figure 4B,C).

First revision

Author response to reviewers' comments

Point-by-point response to reviewers (our reply in blue)

Reviewer 1: SUMMARY OF THE ADVANCE MADE IN THIS PAPER AND ITS POTENTIAL SIGNIFICANCE TO THE FIELD

Almeida et al. report on the relationship between kinetochore size and CENP-E dependence for chromosome alignment in different taxa. Using targeted phylogenetic profiling on CENP-E under mono- versus holocentric conditions, the authors report that this protein was more frequently lost in taxa with holocentric chromosomes. Briefly, analysis in two nematodes revealed that expression of human CENP-E in C. elegans (the CENP-E orthologue is absent in the species) partially rescued chromosome alignment, while CENP-E inactivation in P. pacificus (the CENP-E orthologue is present in the species) had no effect on mitosis.

This paper reports on an interesting aspect of kinetochore biology and the format of a report in the JCS is appropriate for this message to the cell biology community. I have no major points of criticism but I would like the authors to take care of some issues (see below) to improve the presentation of the data.

R: We thank the reviewer for recognizing the interest of our findings and for the suggestions on improving the presentation of the data.

SUGGESTIONS TO AUTHORS

Figures 3 and 4 should be rearranged! The information here is kind of splitted. A jumping from figure 3 to 4 and vice versa is necessary to understand the message. This needs to be avoided. Fig. 3A and Fig. 4A should be combined and shown in one panel. The schematic drawings should be inserted into this new Fig. 3A. In addition, Figure 3C and Figure 4B, and also Figure 3D and Figure 4C should be fused. This will be new Figure 3 B and C. Last, Figure 4D and E would be the

© 2024. Published by The Company of Biologists under the terms of the Creative Commons Attribution License (https://creativecommons.org/licenses/by/4.0/).

new Figure 4A and B.

R: We thank the reviewer for the insight. We have now re-organized the figures according to the reviewer's suggestions. We only note that putting all the information in one single graph looked to us very confusion (shared here for reviewers only) and we decided to split some of the information for the sake of clarity.

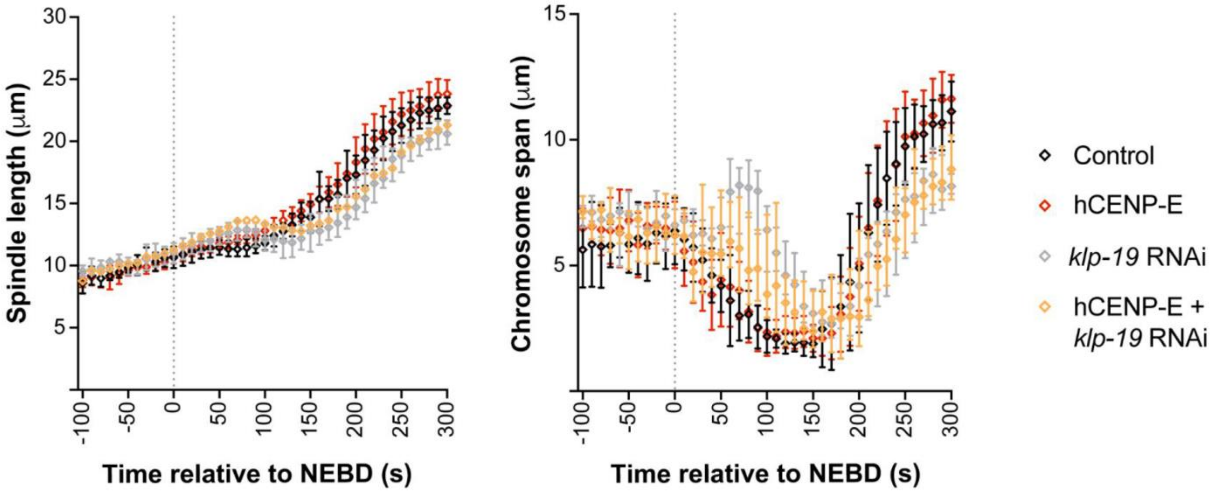

Please make sure to explicitly mention the nematode species (C. elegans or P. pacificus) in the figure legends. This should always be very clear.

R: We now explicitly mention the respective nematode species in the figure legends and included annotations in the figures that allude to the species under study.

I'm not sure about the whole last paragraph of the results section. I realize that a lot of work went into this aspect. In the end, however, what does it really add to this paper. The statement about an effect on the sperm-oocyte switch is very, very general.

R: In this last section of the results we aimed at testing the requirement of CENP-E for mitosis and viability in a nematode system that, contrary to *C. elegans,* encodes a *cenp-E* orthologue. These experiments, when combined with the transgenic expression of human CENP-E in *C.elegans*, confirm the dispensability of CENP-E for mitosis in species with holocentric chromosomes, highlighting that CENP-E might have been retained in *P. pacificus* due to other roles (like the sperm-oocyte switch that determines progeny number) that confer a fitness advantage. We have revised the text to simplify and clarify this important section.

Supplementary Figure 1 needs some explanation and extended information. I think this part of the experiments is not properly explained in the experimental procedures.

R: Supplementary Figure 1 only shows a phylogenetic tree of CENP-E homologs in the selected taxa, rather than results from an experiment. As so, it is merely informative and complementary to the main results. We have described the methodology to generate the depicted phylogeny in full and in accordance to the usual practises for this type of analysis in the methods and figure legend. Nevertheless, we now try to emphasize this analysis further in the main text.

Additional small issue:
Line 77: change "works" to "work".

R: This has been corrected.

Reviewer 2: SUMMARY OF THE ADVANCE MADE IN THIS PAPER AND ITS POTENTIAL SIGNIFICANCE TO THE FIELD

© 2024. Published by The Company of Biologists under the terms of the Creative Commons Attribution License (https://creativecommons.org/licenses/by/4.0/).

The authors of this study follow up on previous work showing that chromosomes with larger kinetochores (in Indian muntjac deer) tend to bi-orient at the metaphase plate without using the motor CENP-E/kinesin-7. Specifically, the authors examine the pattern of CENP-E loss in different animal clades and correlate this with the use of holocentric or monocentric chromosomes. They then carry out some experiments in two nematodes with holocentric chromosomes, one carrying a CENP-E ortholog and one with a CENP-E loss. While the premise is intriguing and the combination of targeted phylogenetic analysis and experiment holds great promise as a general approach to investigate such questions, the study fails to deliver in some critical aspects.

SUGGESTIONS TO AUTHORS

First, the paper (including the title and abstract) claims to investigate the relationship between kinetochore size and CENP-E dependence. However, the authors never measure kinetochore size, neither the variance in average size between species or the variance within species between the different chromosomes. The only comparison is between a small number of holocentric lineages, which exhibit more frequent CENP-E losses, and monocentric lineages, which exhibit fewer losses. While the trend is interesting, the correlation is hardly striking.

R: Although it is indeed true that we do not explicitly measure centromere/kinetochore sizes in these species, which in most cases would be a serious challenge due to the limited availability of resources for the study of these species, we have very carefully checked the available evidence of the holocentric nature of their chromosomes. All the species that we have selected are from well-characterised holocentric lineages. This allows us to subsequently reason that, relative to species with localized centromeres, the kinetochore size of a holocentric species will be generally larger (i.e. covering the entire length of the chromosome), compared to the kinetochore in localized centromeric chromosomes, which is by definition considerably smaller than the overall chromosome size. For instance, kinetochore size in the two nematode species used in our functional studies is over one order of magnitude higher than kinetochores in species with monocentric chromosomes. Therefore, we are confident that a precise measure of kinetochore size in these species is neither feasible in the present work nor essential for our interpretation. Nevertheless, we now adopt a more careful language to refer, as much as possible, to RELATIVE, rather than absolute, kinetochore size throughout the manuscript.

Second, the evolutionary logic presented in this manuscript is not clear. A previous study (with an overlapping author list), Van Hoof et al. EMBO Reports 2017, clearly demonstrates that CENP-E is pan-eukaryotic (with a LECA origin) and in fact greatly expanded through gene duplications in many lineages. Yet on line 91 the authors write: "Altogether, these data raise the exciting possibility that CENP-E and associated, motor-dependent, chromosome alignment pathway, have emerged during evolution to compensate for a reduction in kinetochore size typically observed in vertebrates."

R: The reviewer has a good point here. Indeed, we have incorrectly phrased that conclusion and completely stand by previous work by some of us providing evidence that there is a CENP-E in LECA, suggesting that it was lost (because it was dispensable) in species with holocentric chromosomes. We apologize for overlooking this gross mistake and have now corrected it in the main text.

Third, why is the phylogenetic analysis restricted to CENP-E? Surely it would be worth looking at other components of the "associated, motor-dependent, chromosome alignment pathway"? Are these molecular players known? How many are shared with the motor-independent pathway?

R: We have previously reported based on work in both human and Indian muntjac cells that CENP-E at kinetochores is the critical motor driving the alignment of peripheral chromosomes, dominating over other motors, such as chromokinesins, that assist chromosome alignment but are located on chromosome arms (Barisic et al., NCB, 2014; Drpic et al., Curr Biol, 2018). Our central hypothesis in the present study, based on previous findings in Indian muntjac cells, is that chromosomes with larger kinetochores depend less on CENP-E to align.
Therefore, although we do recognize that other players, such as chromokinesins on chromosome

© 2024. Published by The Company of Biologists under the terms of the Creative Commons Attribution License (https://creativecommons.org/licenses/by/4.0/).

arms, play secondary roles in chromosome alignment, they are not important for our central hypothesis in this manuscript. The reasons underlying our focus on CENP-E are now better explained and justified in the main text.

Fourth, the experiments in the two nematode species do not really report in any way on the relationship between kinetochore size and CENP-E function. It is difficult to conclude anything from the lack of phenotype upon expressing (human) CENP-E in C. elegans - perhaps it is not capable of assuming its normal function within this foreign cellular milieu even if forcibly targeted to kinetochores. (More generally, not much can be confidently inferred from a negative result downstream of in trans expression.) The effect size of the rescue in embryos with attenuated PEF is very small.

R: We would like to draw attention to the fact that the experiment expressing human CENP-E in C. elegans is essentially a control for possible gain of function due to expression of an heterologous protein. The lack of phenotype in a wild-type background is reassuring that this is not the case. The converse experiment in which CENP-E is silenced in P. pacificus with no impact on mitosis further strengthens that CENP-E is dispensable even in holocentric animals where it is normally present. Because chromosome alignment in C. elegans follows a stereotypical and highly reproducible pattern from embryo-to-embryo, it is possible to unveil important phenotypical details that would otherwise go unnoticed in other systems. The fact that expression of human CENP-E does partially rescue chromosome congression defects in C. elegans embryos with attenuated PEFs clearly demonstrates normal function in vivo, when and only if it is necessary. This is now further discussed and clarified in the main text.

Finally, the lack of mitotic phenotypes upon CENP-E inactivation in P. pacificus only shows that there is no obligate dependency relationship in a holocentric species that carries CENP-E - but this would not be expected in any case, especially not given the authors' previous work - as they state in Drpic et al. Current Biology 2018, "Any Chromosome May Use Either the CENP-E-Dependent or -Independent Pathway to Congress, Regardless of Kinetochore Size".

R: The reviewer is correct in concluding that there is no obligate dependency on CENP-E in general, given that alternative/redundant pathways do exist for chromosome alignment. However, it is an overstatement to assume that, for this reason, CENP-E is dispensable. While we stand by our original conclusion that “Any Chromosome May Use Either the CENP-E-Dependent or -Independent Pathway to Congress, Regardless of Kinetochore Size", having a larger kinetochore introduces a bias (not an obligation!) to align independently of CENP-E. This bias, although not essential for chromosome alignment, might represent an advantage in systems that carry CENP-E and have more localized kinetochores. What we test in the present manuscript is whether this advantage can be compensated in systems with holocentric chromosomes due to their relatively large kinetochore size, eventually leading to the loss of CENP-E in some species, or making it totally dispensable for mitosis in species that still carry a CENP-E orthologue. This is now further clarified in the main text.

Thus, overall, I find that the major conclusions of the study, and in particular its central premise, are not really supported by the analyses and experiments presented here, and would urge the authors to include a much more comprehensive phylogenetic analysis supported by experiments that actually measure kinetochore sizes in species selected for experimental follow-up.

R: We are fully aware that our study has limitations in breadth, imposed mostly by the lack of information on absolute kinetochore size in the majority of species analysed. This is now fully acknowledged in the Discussion section in the main text. Nevertheless, we contend that comparing species with holocentric chromosomes in which the kinetochore extends along the entire chromosome arms, with species with regional kinetochores that are often sub-diffraction structures (i.e. less than 200 nm length) is a reasonable first approximation to the problem. We do, however, now refer to previous studies in which kinetochore sizes in the two nematode species used in the present study for experimental follow-up were determined (4 and 2 □m in length in C. elegans and P. pacificus, respectively). Because CENP-E is the critical player in motor-assisted chromosome alignment in species with localized kinetochores (e.g. vertebrates), we believe that extending our phylogenetic analysis to include other genes will add little, if anything, to our conclusions, as they are not relevant for our central hypothesis. We are also

© 2024. Published by The Company of Biologists under the terms of the Creative Commons Attribution License (https://creativecommons.org/licenses/by/4.0/).

strongly convinced that the set of species within the selected taxa already covers the entire diversity of these clades, and adding more species would most likely yield more of the same rather than novel insights (e.g. including extra species in which lineages have lost CENP-E ancestrally will still just have absent CENP-E, as already inferred. Vice-versa, it is unlikely to find CENP-E losses in a clade in which CENP-E is very broadly conserved, as in the case of vertebrates).

Minor:

It is not appropriate to apply t-tests to individual timepoints from continuous time course data (Figure 4B,C).

R: We have redone the statistical analysis to evaluate the overall difference at the critical chromosome alignment phase. Detailed information on our new statistical analysis is now provided in Table S4.

Second decision letter

MS ID#: JOCES/2024/263466

MS TITLE: An evolutionary perspective on the relationship between kinetochore size and CENP-E dependence for chromosome alignment

AUTHORS: Ana C. Almeida; Helder Rocha; Max W. D. Raas; Hanh Witte; Ralf J. Sommer; Berend Snel; Geert J. P. L. Kops; Reto Gassmann; Helder Maiato
ARTICLE TYPE: Short Report

I am happy to tell you that your manuscript has been accepted for publication in Journal of Cell Science, pending standard publication integrity checks.

Reviewer 1

Advance summary and potential significance to field

The authors have properly addressed all my previous comments. From my side, this manuscript should be published in the Journal of Cell Science.

Comments for the author

I have no further suggestions for the authors.

Reviewer 2

Advance summary and potential significance to field

The authors have done their best to address the reviewers' comments within the scope of a reasonable revision, and I appreciate also the adjustments to the text for greater clarity especially on evolution-related arguments. I recommend publication.

© 2024. Published by The Company of Biologists under the terms of the Creative Commons Attribution License (https://creativecommons.org/licenses/by/4.0/).
